# Behavioral Abnormalities in Knockout and Humanized Tau Mice

**DOI:** 10.3389/fendo.2020.00124

**Published:** 2020-03-12

**Authors:** Rafaella Araujo Gonçalves, Nadeeja Wijesekara, Paul E. Fraser, Fernanda G. De Felice

**Affiliations:** ^1^Centre for Neuroscience Studies, Queen's University, Kingston, ON, Canada; ^2^Tanz Centre for Research in Neurodegenerative Diseases, University of Toronto, Toronto, ON, Canada; ^3^Department of Medical Biophysics, University of Toronto, Toronto, ON, Canada; ^4^Department of Psychiatry, Queen's University, Kingston, ON, Canada; ^5^Institute of Medical Biochemistry Leopoldo de Meis, Federal University of Rio de Janeiro, Rio de Janeiro, Brazil

**Keywords:** Alzheimer's disease, MAPT, Tau protein, insulin, anxiety, metabolism, memory

## Abstract

Microtubule-associated protein tau assists in stabilizing microtubules and has been particularly implicated in Alzheimer's disease (AD). Given the importance of tau to AD pathogenesis and therapies, it is important to understand non-classic physiological functions for this protein inside and outside the central nervous system (CNS). Our group has previously shown that tau ablation triggers glucose intolerance and pancreatic dysfunction in mice, suggesting that tau plays a role in peripheral metabolic regulation. Little is known about the role of tau in anxiety. Moreover, inconsistent results have been generated regarding the effects of tau deletion in memory. Here, we characterize systemic insulin resistance, anxiety-related behavior and memory in 15 to 20 weeks old Wild-Type (WT), Tau knockout (TauKO) and a distinct hTau mouse model consisting of tau knockout expressing the longest isoform (2N4R) of a non-mutant WT human Tau protein under the prion promoter (hTau). Our findings demonstrate that tau deletion leads to anxiety-related behavior, impaired contextual and cued fear memory. The presence of a human Tau transgene did not ameliorate the phenotypes observed in animals lacking the mouse tau protein and it elicited impairments in learning, memory, and peripheral insulin sensitivity. Our results suggest that tau protein plays a role in memory and anxiety-related behavior. Our findings also indicate that previously unrecognized functions for tau protein may be a complicating factor in using animal models on the TauKO background. Understanding the link between tau pathophysiology and cognitive and metabolic alterations is of great importance to establish the complete contribution of tau protein to AD pathogenesis.

## Introduction

Tau is a microtubule-associated protein abundant in the Central Nervous System (CNS) with its most well-characterized biological function being microtubules polymerization (1, 2). Hyperphosphorylated tau is the main component of Neurofibrillary tangles (NFT) in Alzheimer‘s Disease (AD) brains (3). In AD, the six Braak stages of the pathology are based on the sequential appearance of NFT in the brain in a hierarchical pattern that correlates with disease severity (4, 5). Tau pathology in the form of NFT correlates with memory loss in normal aging and mild cognitive impairment (MCI) (6, 7). Soluble tau oligomer species isolated from AD patient brains have been implicated in memory impairment, synaptic dysfunction and disease propagation (8–10).

A predominant hypothesis in the AD field is that tau hyperphosphorylation, oligomerization, misfolding, and aggregation into tangles impair synaptic plasticity and contribute to neurodegeneration. These events are probably a result of combined tau gain of toxic function and loss of key physiological function (11, 12). Elucidating unrecognized physiological functions for tau protein is important to better understand the role of this protein in diseases.

The neuropathological spectrum of AD is complex and neuropsychiatric symptoms, particularly depression-related and anxiety, are both reported in patients and considered predictors of disease progression (13–15). Similarly, metabolic alterations are a risk factor and a feature of the AD pathogenesis (16–19). Our group and others have shown that tau ablation triggers glucose intolerance, brain insulin resistance and pancreatic dysfunction in mice, suggesting a physiological role for tau protein in metabolic regulation (20, 21). The effects of tau ablation in mood-related behavior remains to be better elucidated and thus far, inconsistent results have been generated (22–29). Here, we complement our previous metabolic findings on TauKO mouse by investigating the impact of tau loss of function on systemic insulin sensitivity, anxiety-related behavior and memory. We investigate tau gain of toxic function by analyzing the same parameters in a murine Tau knockout mouse expressing the longest isoform of a non-mutant wild type human Tau protein under the prion promoter (hTau).

Our findings show that in the absence of tau, mice develop anxiety-related behavior and memory impairment. Moreover, the insertion of a wild type human tau transgene in TauKO triggered systemic insulin resistance, aggravated memory impairment and did not rescue anxiety phenotype. Notably, hTau present AD-relevant phosphorylation of tau protein and tau oligomers in the neocortex, hippocampus and hypothalamus, when compared to wild type (WT) and TauKO animals.

## Methods

### Animal Care

All experiments were approved by the Animal Care Committee at the University of Toronto. TauKO (B6.129X1-Mapttm1Hnd/J) were purchased from Jackson Labs and have been previously described (30). Mice expressing the longest isoform of the microtubule-associated protein tau (MAPT) gene (2N4R) under the control of cos-tet prion promoter were developed using the same technology as previous described (31). Human Tau expressing mice were crossed with TauKO resulting in hTau/TauKO animals on the C57BL/6 background. C57BL/6 mice are referred to as WT and hTau/TauKO as hTau. TauKO and hTau animals were littermates. Male mice were 15 weeks old at the beginning of the experiments. Euthanasia and tissue collection were performed when animals completed 20 weeks of age.

### Behavioral Analysis

To evaluate anxiety-like behavior and memory, WT, TauKO, and hTau mice were tested for the Open field, Elevated zero maze, Forced swim, Tail suspension, Fear conditioning, Novel object recognition, and Barnes maze behavior tests. Animals were habituated to the testing room at least 1 h prior to testing. The experimenter was blinded to the genotype of the animals for all behavioral studies. All tests were performed between 9:00 and 16:00 in the lights-on cycle. Behavioral effects for each test were observed in at least two independent experiments.

#### Open Field Test

Open field experiments were carried out in an open field arena measuring 0.3 (w) × 0.3 (d) × 0.45 (h) m and divided into nine squares equal in size as previously described (32, 33). During behavioral sessions, each animal was placed at the center of the open field apparatus in which they were allowed to freely explore the empty arena for a 5-min-long session. The time spent exploring the center vs. the periphery of the arena was recorded by a video camera. Total distance traveled and average speed were evaluated to verify possible effects on locomotor exploratory activities.

#### Elevated Zero Maze

The elevated zero maze apparatus consists of an annular platform divided into four equal quadrants: Two opposite enclosed and two opposite opened, as previously described (34). In this study, we used a 50 cm in diameter platform elevated 50 cm above the floor. As previously described (35), during behavioral sessions, each animal was placed on one open arm, facing one of the closed arms of the maze, and was then allowed to freely explore the arena for 5 min. The time spent exploring the closed vs. open arms of the apparatus was recorded by a trained researcher blinded to the genotypes.

#### Forced Swim Test

As previously described (36, 37), one day before the test day, each animal was placed in a 2 L Pyrex glass beaker containing 1,6 L of water at 24 ± 1°C and allowed to freely swim for 15 min. After the 15 min period, the animals were returned to their home cages. On the test day, each mouse was placed individually in a 2 L Pyrex glass beaker containing 1,6 L of water at 24 ± 1°C, for 6 min. A trained researcher blinded to the genotypes recorded immobility time using a stopwatch. The last 4 min of immobility time are plotted in the result graphs. The water was changed between each animal‘s session.

#### Tail Suspension Test

As previously described (38, 39), each animal was suspended from a tape on the tail for 6 min and the immobility time was recorded by a trained researcher blinded to the genotypes.

#### Fear Conditioning

As previously described (40), mice were trained and tested in chambers on three consecutive days in the cued and contextual fear conditioning paradigm. On Day 1, mice were placed into Context A for a total of 180 s. A tone started at the 60th second and lasted for 90 seconds. A 2 s 0.6 mA foot shock was delivered at 88 and 148th s. On Day 2, mice were placed into Context A and were allowed to explore for 300 s without the tone. Freezing was defined as the absence of movement except that which is required for respiration. On Day 3, mice were placed into Context B and were allowed to explore for 300 s. The tone started at the 120th s and lasted for 180 s. Fear memory for the context (contextual memory) or the tone (cued memory) was obtained by calculating the percentage of freezing on day 2 or 3, respectively. Freezing behavior was recorded by measuring beam breaks in 1 s intervals and analyzed using Freeze Monitor (San Diego Instruments). In our analysis, we set a threshold of two beam breaks to be considered as movement.

#### Novel Object Recognition Test

As previously described (41, 42), object recognition experiments were carried out in an open field arena measuring 0.3 (w) × 0.3 (d) × 0.45 (h) m^3^. Test objects were made of plastic and had different shapes, colors, sizes and textures. During behavioral sessions, objects were fixed with tape to the floor so that the animals could not move it. None of the objects used in our experiments evoked innate preference. Before training, each animal was submitted to a 5-min-long habituation session, in which they were allowed to freely explore the empty arena. Training consisted of a 5-min-long session during which animals were placed at the center of the arena in the presence of two objects. The time spent exploring each object was recorded by a trained researcher. Sniffing and touching the object were considered as exploratory behavior. The arena and objects were cleaned thoroughly between trials with 50% alcohol (vol/vol) to ensure minimal olfactory cues. One hour after training, animals were reinserted into the arena for the test session, when one of the two objects used in the training session was replaced by a new one. The time exploring familiar and novel objects were measured. Results were expressed as percentage of time exploring each object during the training or test sessions.

#### Barnes Maze

As previously described (43), the Barnes Maze paradigm consists of an elevated and circular platform with 18 equally spaced holes. Under one of the holes, named ‘target‘, a small dark recessed chamber was positioned which the animals could access to escape from the platform. Bright light was used as the aversive stimuli. Visual cues were placed surrounding the maze. Learning, short and long-term memory retention were evaluated. The test consisted of: i. Adaptation period: The animals were placed in the middle of the maze in a cylindrical black chamber for 10 s and then were gently guided to the target hole with the aversive stimuli on. Once the animals reached the target hole, the aversive stimuli were turned off and the animals were kept inside the escape box for 2 min. ii. Spatial acquisition: The animals were placed in the middle of the maze in a cylindrical black chamber for 10 s and then were allowed to freely explore the maze for 3 min with the aversive stimulus on. Primary errors, total errors, primary latency, total latency, were measured by the experimenter. When the animal reached the target hole or when 3 min had elapsed, the mouse was allowed to stay in the target box for 1 min. Animals received 4 trials per day for 4 days, with an inter-trial interval of 15 min. iii. Probe day (short- and long-term memory retention): On day 5 and on day 12, 24 h and 8 days after the last training day, respectively, the probe trials were conducted. The target hole was closed, and the animals were allowed to explore the maze for 90 s. Number of pokes (errors) and latency to reach the virtually target hole, were measured.

### Immunoblot Analyses

Mice were euthanatized and the neocortex, hippocampus and hypothalamus were rapidly dissected and frozen in dry ice. For total protein extraction, samples were homogenized in RIPA lysis buffer containing protease and phosphatase inhibitors. Protein concentration was determined using the Pierce BCA Protein Assay Kit. Aliquots containing 20 ug of protein were resolved in 4–20% Mini-PROTEAN TGX Precast Protein Gels (Bio-Rad) and were eletrotransferred to nitrocellulose membranes for 90 min at 100 V. Blots were blocked for 1 h with 10% skim milk in TBS-T at room temperature and were incubated overnight at 4C with primary antibodies diluted in TBS-T. 10 uL of molecular weight markers were run in one lane in every gel (Precision Plus Protein Kaleidoscope, Bio-Rad). The primary antibodies used were the rabbit polyclonal anti-Tau AB0024 (1:100000; DAKO), rabbit polyclonal anti-Tau oligomer T22 (1:100000; Millipore), rabbit polyclonal anti-TauSer199Ser202 (1:1000; ThermoFisher) and mouse monoclonal anti-beta Actin (1:10000; Abcam). After incubation with primary antibodies, membranes were incubated with horseradish peroxidase-conjugated secondary antibody (anti-mouse or anti-rabbit; 1:5000) diluted in TBS-T at room temperature for 2 h. Chemiluminescence was detected using ECL substrate (Amersham) for 5 min and imaged using Azure Biosystems.

### Insulin and Leptin Enzyme-Linked Immunosorbent Assay (ELISA)

After 4 h fast, blood was collected in EDTA-coated microvettes (Sarstedt) from tail vein and plasma was isolated. Insulin levels from brain lysates (neocortex, hippocampus, and hypothalamus) and plasma were measured using ultrasensitive insulin ELISA (ALPCO Diagnostics). Leptin levels were measured using a Mouse Leptin ELISA Kit (Crystal Chem).

### Insulin Tolerance Test

Following 4 h fast, insulin (1 IU/kg body weight) was injected intraperitoneally and plasma glucose was measured at 0, 15, 30, and 60 from tail vein blood using a glucometer.

### Statistical Analysis

Values are expressed as means +/– SEM. Significance was determined using Student's *t*-test or one-way ANOVA followed by Holm-Sidak *post-hoc* test. All analyses were performed with GraphPad Prism6® (GraphPad Software).

## Results

### Peripheral Insulin Sensitivity and Brain Insulin Levels of TauKO and hTau Mice

**Figure 1:**
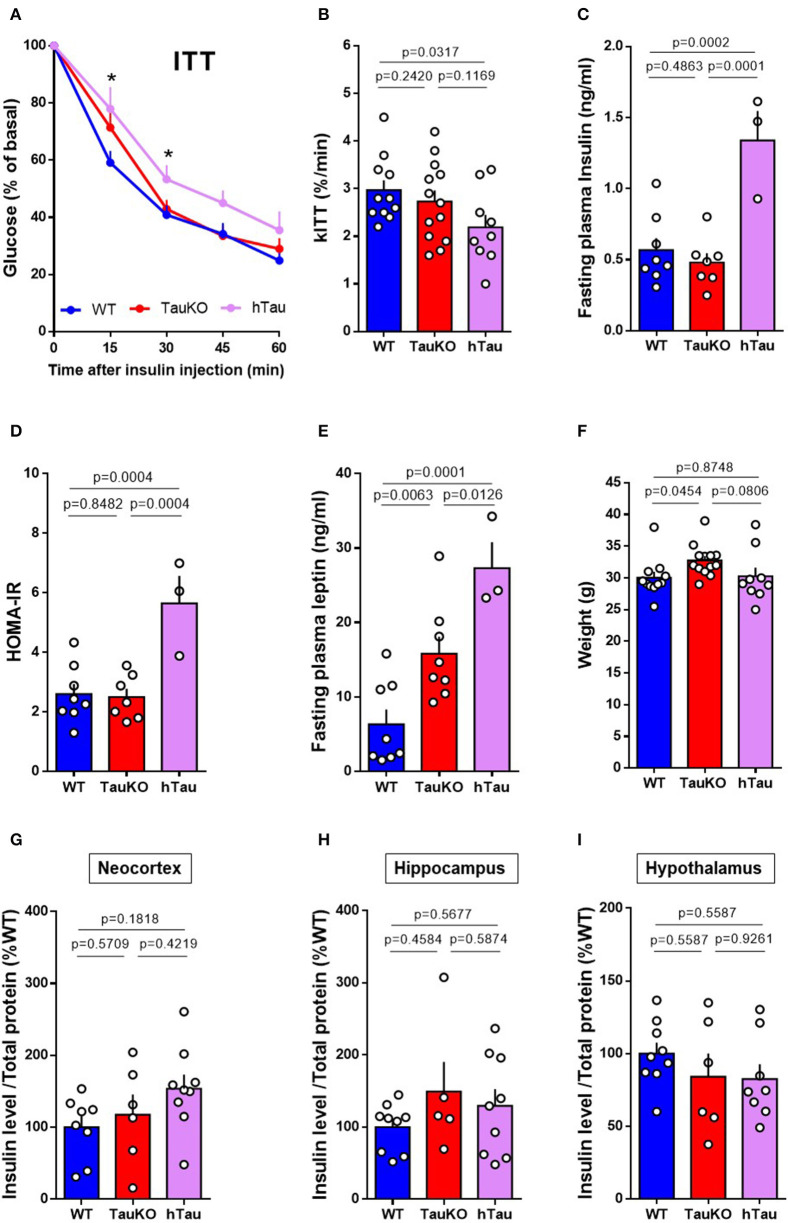
Peripheral insulin sensitivity and brain insulin levels of TauKO and hTau mice. **(A)** Insulin Tolerance Test (ITT) with 20 weeks old WT, TauKO, or hTau mice. After 4 h fasting, mice received 1U/kg of intraperitoneal insulin and blood glucose levels were measured at the designated time points from tail vein blood (*n* = 11 WT; 13 TauKO; 9 hTau). **(B)** Bar graphs representing the kinetic constants for glucose disappearance (Kitt) calculated from the time course plot (*n* = 11 WT; 13 TauKO; 9 hTau). **(C)** Plasma insulin levels after fasting measured by ELISA (*n* = 8 WT; 7 TauKO; 3 hTau). **(D)** HOMA-IR calculated from glucose (mMol/L) and insulin (mU/L) levels, using the formula: HOMA = fasting glucose (mMol/L) x fasting insulin (mU/L)/22.5 (*n* = 8 WT; 7 TauKO; 3 hTau). **(E)** Plasma leptin levels after fasting measured by ELISA (*n* = 8 WT; 7 TauKO; 3 hTau). **(F)** Body weight (*n* = 11 WT; 13 TauKO; 9 hTau). **(G–I)** Levels of insulin in lysates from the neocortex (*n* = 8 WT; 6 TauKO; 9 hTau), hippocampus (*n* = 9 WT; 5 TauKO; 9 hTau), and hypothalamus (*n* = 9 WT; 6 TauKO; 8 hTau), measured by ELISA. Data are representative of two independent experiments. *p < 0.5.

Tau ablation in mice leads to pancreatic beta cell dysfunction and glucose intolerance (20, 21). In agreement with our previous study (21), here we show that Tau deletion does not affect systemic insulin sensitivity in 20 weeks old mice. WT and TauKO did not show differences in the percentage of blood glucose reduction after intraperitoneal injection of insulin during the Insulin Tolerance Test (ITT) (Figures 1A,B). Fasting plasma insulin levels (Figure 1C) and HOMA-IR index (Figure 1D) also did not differ between WT and TauKO mice. Surprisingly, the insertion of a transgene that encodes the longest isoform of human Tau (2N4R) triggered insulin resistance in TauKO animals. hTau mice displayed insulin resistance in the ITT (Figures 1A,B), increased fasting plasma insulin levels (Figure 1C) and higher HOMA-IR index (Figure 1D) when compared to WT and TauKO.

Augmented body weight and hyperleptinemia were previously reported following tau ablation in mice (20, 21). Interestingly, although in our current study the hTau transgene aggravates hyperleptinemia (Figure 1E), hTau expression seems to correct the increase in body weight resulted from tau deletion (Figure 1F). Therefore, hyperleptinemia in hTau mice might result from other factors than increased fat mass.

Brain insulin has been implicated in the modulation of metabolism and neurobehavior in rodents (44, 45). Moreover, tau ablation promotes insulin resistance in the brain of mice (20). To investigate whether insulin levels were altered in the brains of TauKO and hTau, the levels of insulin in the neocortex (Figure 1G), hippocampus (Figure 1H) and hypothalamus (Figure 1I) were determined by ELISA. However, no statistical differences were observed between the experimental groups.

In summary, our results show that the presence of a hTau transgene impairs peripheral insulin sensitivity and systemic leptin levels at 20 weeks of age, without affecting insulin levels in different brain regions.

### Patterns of Anxiety-Related Behaviors in TauKO and hTau Mice

**Figure 2:**
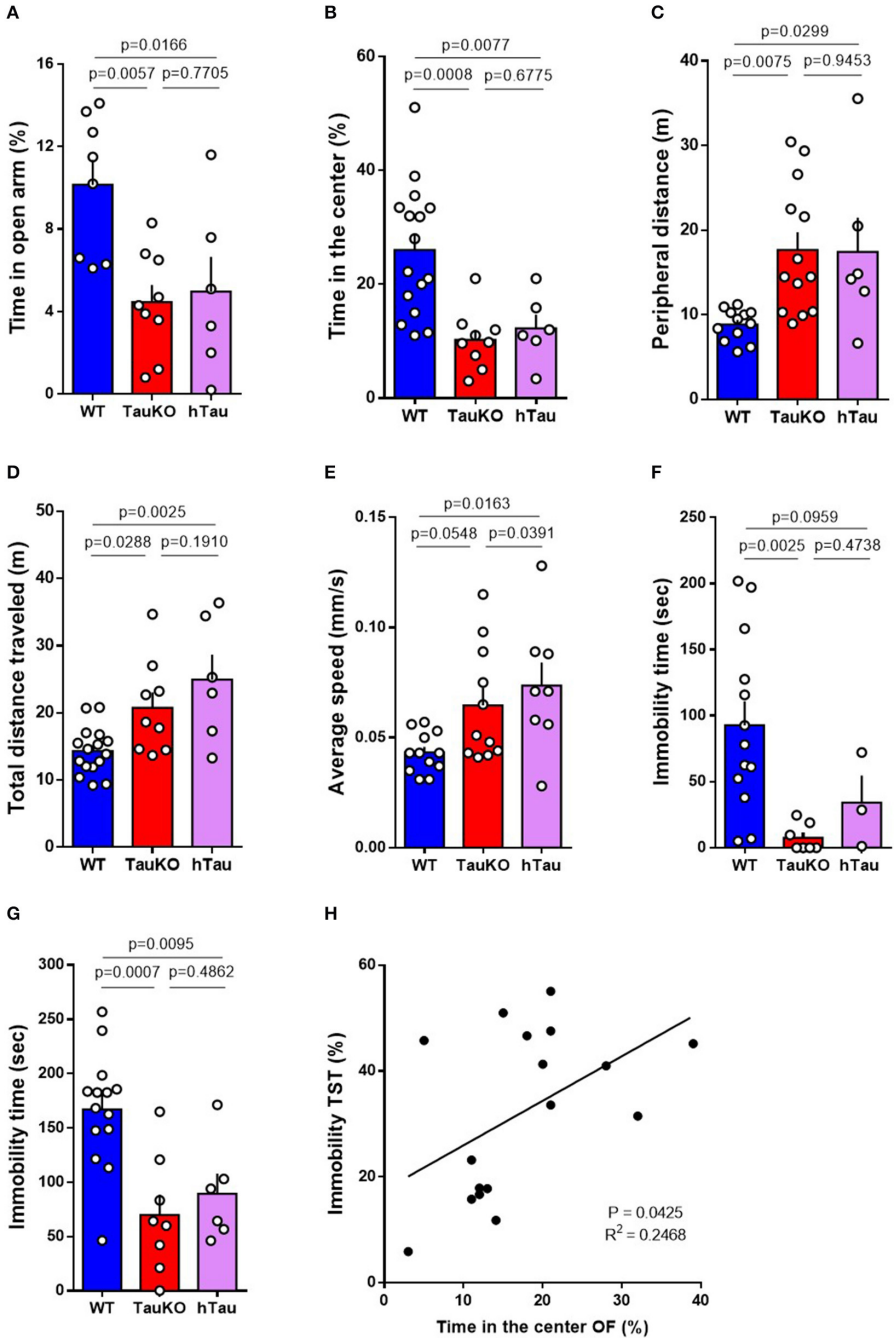
Patterns of anxiety-related behaviors in TauKO and hTau mice. **(A)** Analysis of the time spent in the open arms of the elevated zero maze expressed in percentage of time relative to the 5 min' test length. Test performed with 15–19 weeks old WT, TauKO, and hTau mice (*n* = 8 WT; 9 TauKO; 6 hTau). **(B–E)** Open field test performed with 15–19 weeks old WT, TauKO, and hTau mice (*n* = 12–16 WT; 9–13 TauKO; 6–8 hTau). **(B)** Analysis of the time spent in the center of the apparatus expressed in percentage of time relative to the 5 min' test length. **(C)** Analysis of the total distance moved in the periphery of the apparatus. **(D)** Analysis of the total distance traveled in the whole apparatus. **(E)** Analysis of the average speed during the test. **(F)** Time of immobility during the forced swim test performed with 15–19 weeks old WT, TauKO, and hTau mice (*n* = 13 WT; 7 TauKO; 3 hTau). **(G)** Time of immobility during the tail suspension test performed with 15–19 weeks old WT, TauKO, and hTau mice (*n* = 14 WT; 8 TauKO; 6 hTau). **(H)** Correlation of immobility time during the TST and time spent in the center of the OF for each mouse. Values expressed in percentage of time relative to the total length of the tests. Data are representative of at least two independent experiments.

Impaired metabolic regulation is associated with anxiety symptoms (46, 47). Therefore, we investigated anxiety-related behavior in 15–19 weeks old WT and TauKO mice at the open field (OF), elevated zero maze (EZM), forced swim, and tail suspension behavior tests. TauKO spent significantly less time in the open arms of the EZM (Figure 2A), and in the central area of the OF apparatus (Figure 2B), when compared to WT animals. In addition to that, TauKO moved more in the periphery of the OF arena (Figure 2C). The reduced time exploring the center of the OF and open arms of the EZM indicate higher anxiogenic behavior in TauKO when compared to WT mice (33, 35). Tau ablation also affected the locomotor exploratory activity of mice during the OF test indicated by increased total ambulatory distance (Figure 2D) and average speed (Figure 2E). Unexpectedly, the insertion of a human tau transgene did not correct anxiety-related behaviors of TauKO animals. Similar to TauKO, hTau mice spent less time in the open arms of the EZM and in the center of the OF apparatus (Figures 2A,B), exhibited increased peripheral (Figure 2C) and total ambulatory distance (Figure 2D), and augmented average speed (Figure 2E).

The tail suspension (TST) and forced swim (FST) tests are commonly used for the detection of behavior despair in mice and for the screening of antidepressants (48). In this context, increased immobility time indicates depressive-like behavior (36, 39). However, studies have suggested the use of TST and FST for the detection of anxiety-related behavior arguing that reduced immobility time in these tests results from exacerbated escape-directed behavior caused by an anxiogenic phenotype (49). Here, we report reduced immobility time of 15–19 weeks old TauKO and hTau mice in the FST (Figure 2F) and TST (Figure 2G) when compared to WT animals. We believe that these results do not represent an antidepressant-like response but is instead caused by the anxiogenic effect of knocking out Tau that is not corrected by the addition of the hTau transgene in the hTau mice. In agreement with this idea, linear regression analysis showed a positive correlation between immobility time in the TST and time spent in center of the OF arena (Figure 2H). Similar to previous reports (50, 51), we observed an inverse relationship between behavior despair and anxiety-related behavior in our mouse cohort. In summary, our results indicate that whole-body murine tau deletion leads to anxiety-related behavior that is not corrected by the presence of a human tau transgene.

### Performance in the Fear Conditioning, Novel Object Recognition and Barnes Maze Behavior Tests

Poor glycemic control is associated with cognitive decline (52, 53). Therefore, to determine the effect of tau deletion on memory, 15–19 weeks old WT and TauKO mice were subjected to the Fear conditioning (FC), Novel object recognition (NOR) and Barnes maze tests.

**Figure 3:**
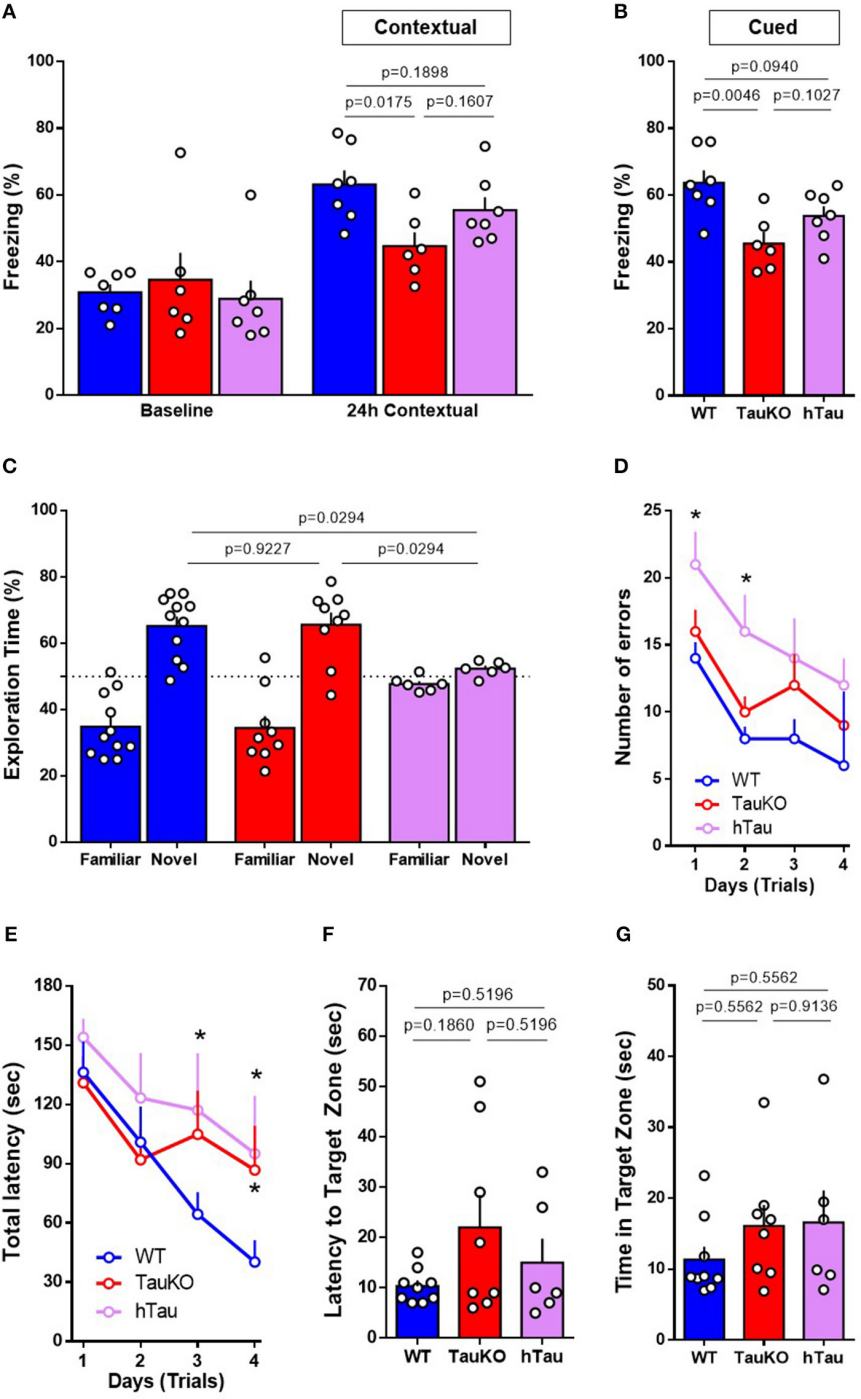
Performance in the Fear Conditioning, Novel Object Recognition and Barnes Maze behavior tests. **(A,B)** Fear conditioning test performed with 15–19 weeks old WT, TauKO, and hTau mice (*n* = 7 WT; 6 TauKO; 7 hTau). Percentage of freezing behavior during the **(A)** contextual and **(B)** cued FC. **(C)** Novel object recognition test performed with 15–19 weeks old WT, TauKO, and hTau mice (*n* = 11 WT; 9 TauKO; 6 hTau). Exploration time of familiar and novel objects during the test phase. Time expressed in percentage. **(D–G)** Barnes maze test performed with 15–19 weeks old WT, TauKO, and hTau mice (*n* = 9 WT; 8 TauKO; 6 hTau). **(D)** Total number of errors and **(E)** Total latency to find the target zone in 4 different trials/day, for 4 days during the acquisition/training phase of the Barnes maze test. **(F)** Latency to find and **(G)** time in the target zone 24 h after the last training trial as a measure of spatial reference memory. Data are representative of at least two independent experiments. *p < 0.5.

Fear conditioning was performed to assess hippocampal and amygdala learning and memory. In contextual fear conditioning, TauKO mice displayed reduced freezing behavior when compared to WT animals (Figure 3A). In cued fear, when animals were placed in a new context but with the same auditory conditioned stimulus, TauKO showed a more pronounced reduction in freezing than WT animals (Figure 3B). Similarly, hTau mice had reduced freezing when compared to WT in both tests.

The novel object recognition (NOR) test involves several brain regions of learning and memory. During the acquisition/training phase, WT, TauKO and hTau mice were exposed to two objects for 5 min and no object preference was observed (Figure S1A). After 1-h interval, each animal was individually reintroduced to the apparatus and allowed to explore one familiar and one novel object for 5 min. By definition, animals that recognize the familiar object (i.e., normal learning) explore the novel object for a time significantly higher than 50% of the total time. Our results show that while WT and TauKO mice correctly discriminated between familiar and novel object (Figure 3C), hTau mice displayed impaired object recognition memory indicated by the lack of preference for the novel object over the familiar one (Figure 3C). Total exploration time and time exploring the new object did not exhibit statistically significant differences for the various test groups (Figures S1B,C).

Lastly, Barnes maze was performed to assess non-hippocampal contributions to spatial memory. During the acquisition training phase of the test, while WT and TauKO mice displayed similar total latency and errors to enter the target hole during the trials, hTau mice showed impaired learning indicated by increase in total errors and total latency (Figures 3D,E). The latency to find the target zone (Figure 3F) and the time spent in this area 24 h (Figure 3G) after the last training trial, probe day 5, did not differ between the experimental groups. Histograms representing the mean number of nose pokes in each hole of the Barnes Maze during the probe day 5 are depicted (Figure S2A) and no statistical difference was detected regarding the % of pokes in the target hole (*p* = 0.7537 between WT and TauKO; *p* = 0.6480 between WT and hTau; *p* = 0.7537 between TauKO and hTau. One-way ANOVA). Similar results were observed when these parameters were analyzed 8 days after the last training trial, probe day 12 (Figures S2B–D). Primary errors (Figure S2E) and primary latency (Figure S2F) were also similar between the experimental groups.

In summary, our results indicate that Tau deletion leads to defective associative fear memory in mice that is not corrected by a hTau transgene. On the other hand, object recognition and spatial memory are not affected by tau ablation and are impaired after the addition of a hTau transgene in the hTau mice.

### Phospho-Tau and Tau Oligomers Are Increased in Multiple Brain Regions of hTau Mice

**Figure 4:**
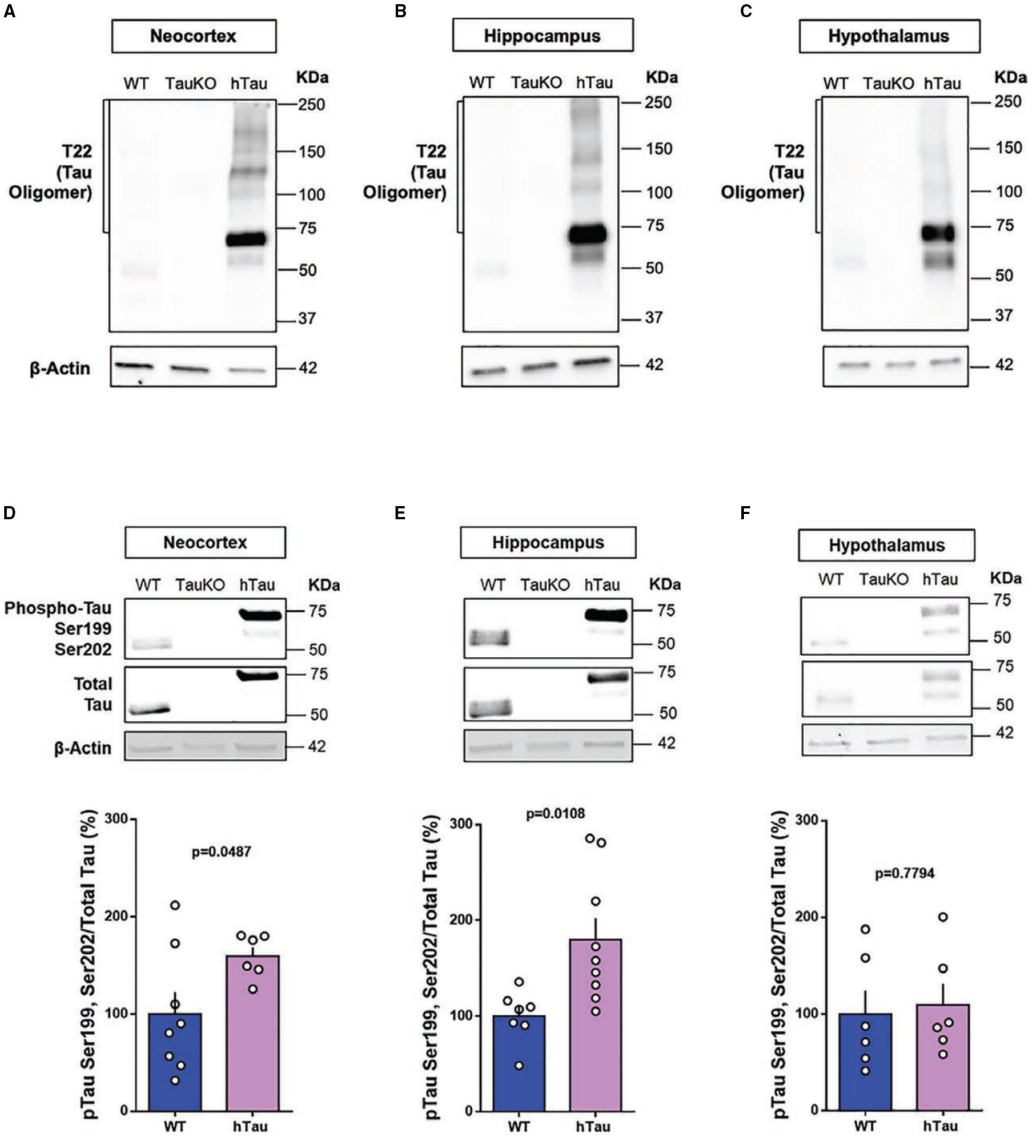
Phospho-tau and tau oligomers are increased in multiple brain regions of hTau mice. Immunoblot analysis of tau oligomers in **(A)** cortical, **(B)** hippocampal, and **(C)** hypothalamic lysates from 20-week-old WT, TauKO, and hTau mice. Immunoblot analysis of pTauSer199Ser202/TotalTau ratio in **(D)** cortical (*n* = 8 WT; 6 hTau), **(E)** hippocampal (*n* = 7 WT; 9 hTau), and **(F)** hypothalamic (*n* = 6 WT; 6 hTau) lysates from 20-week-old WT, TauKO, and hTau mice.

Tau oligomers and pTauSer199Ser202 are detected in *post-mortem* AD brains and are believed to play a role in AD pathophysiology (54, 55). Therefore, to investigate a possible gain of toxic function for tau as an underlying mechanism for the behavioral alterations observed in hTau mice, we investigated the presence of AD-relevant phosphorylation of tau protein and tau oligomers in the neocortex, hippocampus and hypothalamus of hTau mice. Immunoblotting results targeting total tau protein confirmed the absence of tau in the TauKO mice and the presence of tau in the neocortex, hippocampus and hypothalamus of 20 weeks old WT and hTau mice (Figure 4). Tau Oligomers (Figures 4A–C) and phosphorylated tau at the Serine-202 and Serine-199 residues (Figures 4D–F) were detected in the neocortex, hippocampus and hypothalamus of hTau mice by immunoblotting analysis. Intriguingly, while phospho-tau levels were elevated in the neocortex and hippocampus of hTau mice when compared to WT animals, no changes were observed in the hypothalamus. Conversely, tau oligomers were consistently elevated in the different brain regions.

## Discussion

The present study provides evidence for a role of tau protein in anxiety and memory. Tau ablation in mice led to anxiety-related behavior and memory impairment. We also showed that the insertion of a human tau transgene in TauKO mice triggered peripheral insulin resistance, aggravated memory impairment and did not ameliorate anxiety phenotype in 15–20 weeks old animals.

Tau protein was first described in 1975 as a factor essential for microtubule assembly and polymerization in the porcine brain (1). In 1986, hyperphosphorylated tau was identified as one the main constituents of neurofibrillary tangles in the AD brain (3). The timeline of the scientific discoveries involving tau led to investigations on the role of this protein in the brain and in diseases. However, tau is expressed in a variety of tissues (56–58) and understanding unknown physiological functions for this protein inside and outside the CNS is key to understand the role of tau in the pathophysiology of diseases. Although tau deletion is not lethal (59), tau knockout mice have been instrumental to the understanding of novel functions for tau protein in physiology and in pathology (60).

Metabolic syndrome and insulin resistance are associated with cognitive dysfunction and AD (53, 61–65). Therefore, because of our previous publication showing impaired glucose-stimulated insulin secretion in TauKO and hTau mice (21), and our recent finding demonstrating insulin resistance in hTau animals, we initially hypothesized that the levels of insulin getting to the brain of these mouse models would be reduced. However, we did not find statistical differences in the levels of insulin in the neocortex, hippocampus and hypothalamus between the experimental groups. Considering the proposed role for tau protein in the regulation of insulin signaling in the hippocampus (20), it is likely that the behavioral alterations of TauKO and hTau result from impaired insulin signaling instead of reduced hormonal levels in the brain. Further studies aiming to investigate insulin signaling pathway in different brain regions of hTau mice are warranted.

Anxiety is reported in up to 75% of AD patients (66) and it is associated with increased rates of conversion from MCI to AD (14). Here, we show that 15–19 weeks old TauKO and hTau mice displayed anxiety-related behavior in the elevated zero maze, open field, forced swim and tail suspension tests. Hyperactivity was also observed, characterized by increased total ambulatory locomotion and average speed in the OF arena. Interestingly, a positive correlation was found between the time spent in the center of the OF apparatus and the immobility time during the tail suspension test. As suggested by a recent commentary (49), and in validation of our correlational results, we believe that the reduced immobility in the FST and TST is a measure of anxiety behavior/hyperactivity rather than an antidepressant effect of tau deletion in mice. The mechanisms underlying the increase in anxiety-like behavior following tau ablation in mice are unknown and may involve several mechanisms. Conversely, other studies did not detect an anxiogenic phenotype in 6 or 7-months-old TauKO mice in the zero maze (29) and open field (26) behavior tests. These differences in results might be due to the age, strain and sex of the animals, as well as behavior test protocol used in each study.

Impaired metabolic regulation is associated with anxiety behavior (46, 47). Therefore, it is possible that the severe glucose intolerance in TauKO and hTau mice (21) is involved in the increase of anxiety-related behavior reported in this study. Moreover, leptin is an adipokine-derived hormone upregulation of which triggers anxiolytic phenotype in mice (67) and deficiency results in anxiogenic-like behavior (68, 69). Plasma leptin levels are associated with the emotional state of individuals throughout the day (70) and serum leptin and leptin resistance correlated with anxiety symptoms in patients with type 2 diabetes (T2D) (71). Therefore, the augmented circulating leptin levels reported in TauKO and hTau mice (Figure 1E) could be involved in the anxiogenic phenotype observed in these animals, probably as a consequence of leptin resistance following chronic hyperleptinemia.

The dopaminergic system plays an important role in the modulation of anxiety behaviors (72). The enzyme tyrosine hydroxylase (TH) catalyzes the reaction of L-DOPA formation, which precedes its conversion in dopamine. In line with that, although one study reported mild dopaminergic deficits in aged but not in adult TauKO mice (24), other investigators have showed reduced TH-positive nigral neurons and decreased TH expression in the substantia nigra of adult TauKO animals (22, 73). Therefore, it is possible that tau ablation impacts the homeostasis of the dopaminergic system resulting in anxiety-behavior in mice.

Memory loss is a widely known clinical manifestation of AD. However, conflicting results have been generated regarding the impact of tau deletion on memory in mice (22–29). In this study, we investigated memory integrity using three distinct behavior tests. Fifteen to nineteen weeks old TauKO mice showed impaired associative learning in the fear conditioning (FC) paradigm and no alterations in object recognition or spatial and learning memory in the novel object recognition (NOR) and Barnes maze, respectively. Impaired performance in the FC test is associated with defective hippocampal and amygdala function (74, 75), while memory defects in the NOR task are sensitive to cortical injuries (76). Therefore, our results suggest that tau ablation in mice differently impacts memory-related brain regions.

Tau protein plays a role in the regulation of synaptic function (11). Accordingly, a role for tau in synaptic plasticity has been suggested by reports showing deficits in hippocampal long-term potentiation (LTP) and/or long-term depression (LTD) (26, 29, 77), as well as reduced hippocampal brain-derived neurotrophic factor (BDNF) levels following tau deletion or knockdown in mice (22, 78). Therefore, impairments in synaptic plasticity might be involved in the behavioral alterations reported in TauKO and hTau, in this study.

Tau is an intrinsically unfolded and soluble protein that undergoes a number of post-translational modifications that affect its structure and therefore its function inside the cells. Among these modifications, hyperphosphorylation has been implicated in the pathogenesis of AD due to its capability of affecting tau self-assembly, aggregation and its accumulation into Neurofibrillary tangles (NFT) (3). The phosphorylation of tau at Ser199/Ser202 is particularly increased in human AD brains (54) and tau oligomers were isolated from the brains of patients (8, 55). The cerebral accumulation of soluble small oligomeric tau species correlates with neuronal loss, synaptic dysfunction and behavior alterations associated with AD (9, 10). Therefore, in addition to the physiological function of tau in synaptic activity, pathological tau can induce synaptic damage in AD.

In conjunction with the behavioral changes, elevated tau oligomers were observed in cognitive-related (neocortex and hippocampus) and metabolic-related (hypothalamus) brain regions of hTau mice. Increased tau phosphorylation at Serine-199 and Serine-202 residues were also detected in the neocortex and hippocampus of these animals. Therefore, toxic gain of tau function in hTau mice might be elicited by the phosphorylated and oligomeric tau species detected in these animals. In terms of loss of function, because tau isoforms differ between humans and rodents (79), human tau might not compensate for the absence of endogenous murine tau in the humanized mice. Therefore, an imbalance in the tau isoforms expressed in different brain and peripheral tissues of hTau mice might explain the lack of compensation of this model.

**Figure 5:**
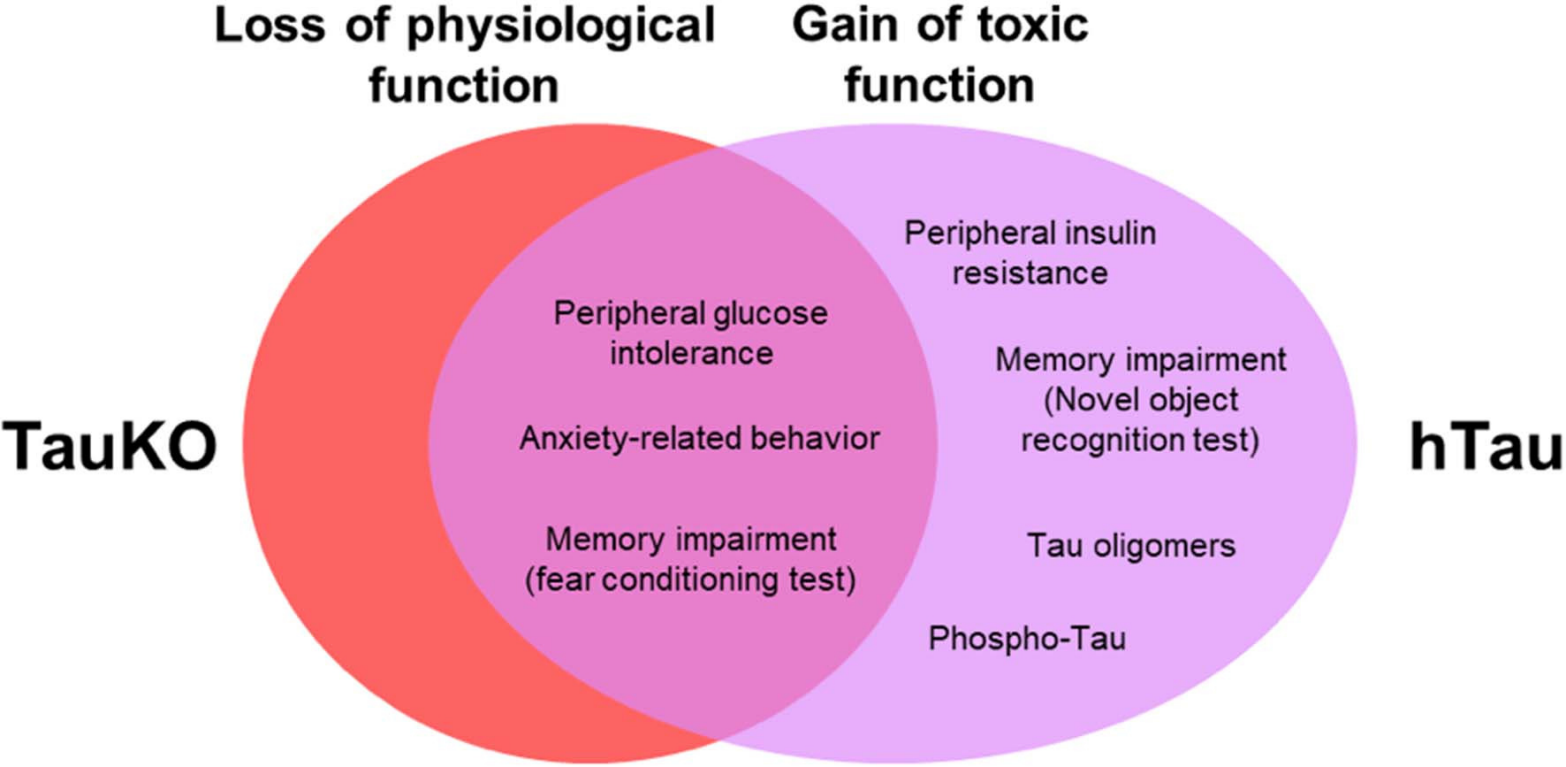
Behavioral and metabolic alterations in TauKO and hTau mice. Here, we hypothesize that tau loss of function in the knockout, and a combined loss and gain of toxic function for tau in the hTau mice, underlie the behavioral and metabolic alterations observed in both mouse models in this study.

Collectively, our results from TauKO mice suggest a physiological role for tau in anxiety-related behavior and memory. Results from hTau mice demonstrate that the presence of a non-mutant WT human tau triggers insulin resistance, elicit impairments in spatial learning and object recognition memory, and does not restore anxiety, memory and metabolic alterations in mice lacking endogenous murine tau (Figure 5). Our findings also suggest that previously unrecognized functions for tau protein is a potentially complicating factor in using animal models on the TauKO background. Understanding the link between tau pathophysiology, and cognitive and metabolic alterations is of great importance to establishing the complete contribution of tau protein to AD pathogenesis.

## Data Availability Statement

The datasets generated for this study are available on request to the corresponding author.

## Ethics Statement

The animal study was reviewed and approved by the Local Animal Care Committee (LACC) at the University of Toronto. Animal Use Protocol number 5832.

## Author Contributions

RG designed the experiments, acquired, analyzed, and interpreted the data, and drafted the manuscript. NW contributed to the acquisition and interpretation of data. FD and PF provided substantial contributions to the conception of the study, experimental design, and data interpretation. All authors revised and approved the final version of the manuscript.

### Conflict of Interest

The authors declare that the research was conducted in the absence of any commercial or financial relationships that could be construed as a potential conflict of interest.

## Abbreviations

AD: Alzheimer's disease
BDNF: Brain-derived neurotrophic factor
CNS: Central nervous system
ELISA: Enzyme-linked immunosorbent assay
EZM: Elevated zero maze
FC: Fear Conditioning test
FST: Forced swim test
ITT: Insulin Tolerance Test
LepR: Leptin receptor
LTD: Long-term depression
LTP: Long-term potentiation
MAPT: Microtubule-associated protein tau
MCI: Mild cognitive impairment
NFT: Neurofibrillary tangles
NOR: Novel Object Recognition test
OF: Open Field
TauKO: Tau knockout
TH: Tyrosine hydroxylase
TST: Tail suspension test
WT: Wild type.
