# Erratum: Behavioral Abnormalities in Knockout and Humanized Tau Mice

**DOI:** 10.3389/fendo.2021.804873

**Published:** 2021-11-18

**Authors:** Frontiers Production Office

**Affiliations:** Frontiers Media SA, Lausanne, Switzerland

**Keywords:** Alzheimer’s disease, MAPT, Tau protein, insulin, anxiety, metabolism, memory

Due to a production error, there was a mistake in **Figure 4A** as published. Image 4A has a blank box, containing no data. The corrected **Figure 4A** appears below.

**Figure 4:**
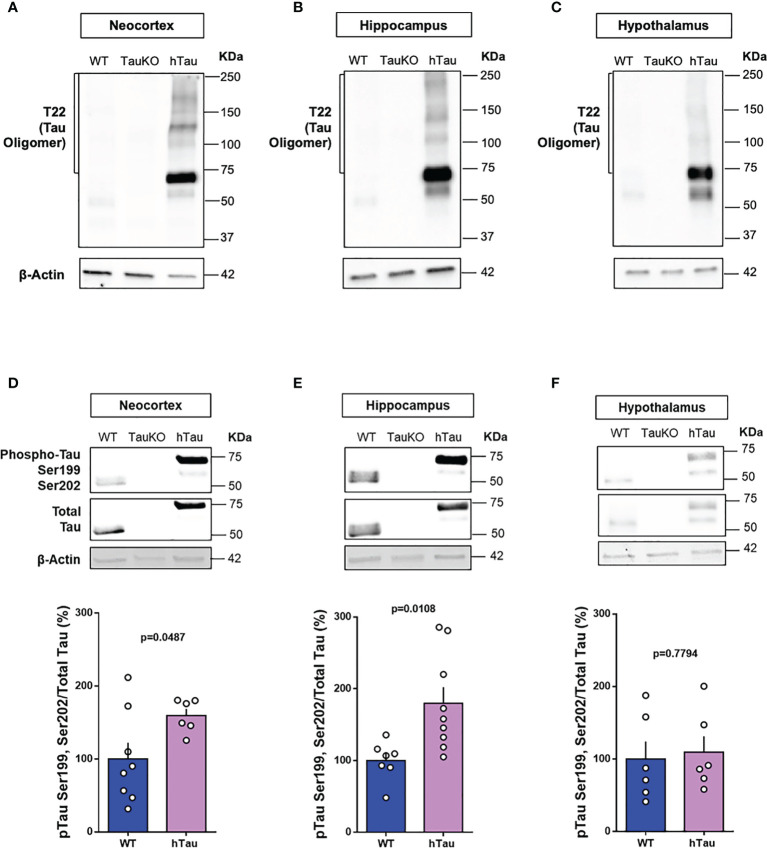
Phospho-tau and tau oligomers are increased in multiple brain regions of hTau mice. Immunoblot analysis of tau oligomers in **(A)** cortical, **(B)** hippocampal, and **(C)** hypothalamic lysates from 20-week-old WT, TauKO, and hTau mice. Immunoblot analysis of pTauSer199Ser202/TotalTau ratio in **(D)** cortical (n= 8 WT; 6 hTau), **(E)** hippocampal (n = 7 WT; 9 hTau), and **(F)** hypothalamic (n = 6 WT; 6 hTau) lysates from 20-week-old WT, TauKO, and hTau mice.

The publisher apologizes for this mistake.

The original version of this article has been updated.

