# Supplementary material for: Behavioral Abnormalities in Knockout and Humanized Tau Mice
Source: Front Endocrinol (Lausanne). 2020 Mar 12;11:124. doi: 10.3389/fendo.2020.00124 (PMC7080660; doi:10.3389/fendo.2020.00124)
Supplement: Supplementary file 1 [file Data_Sheet_1.pdf]

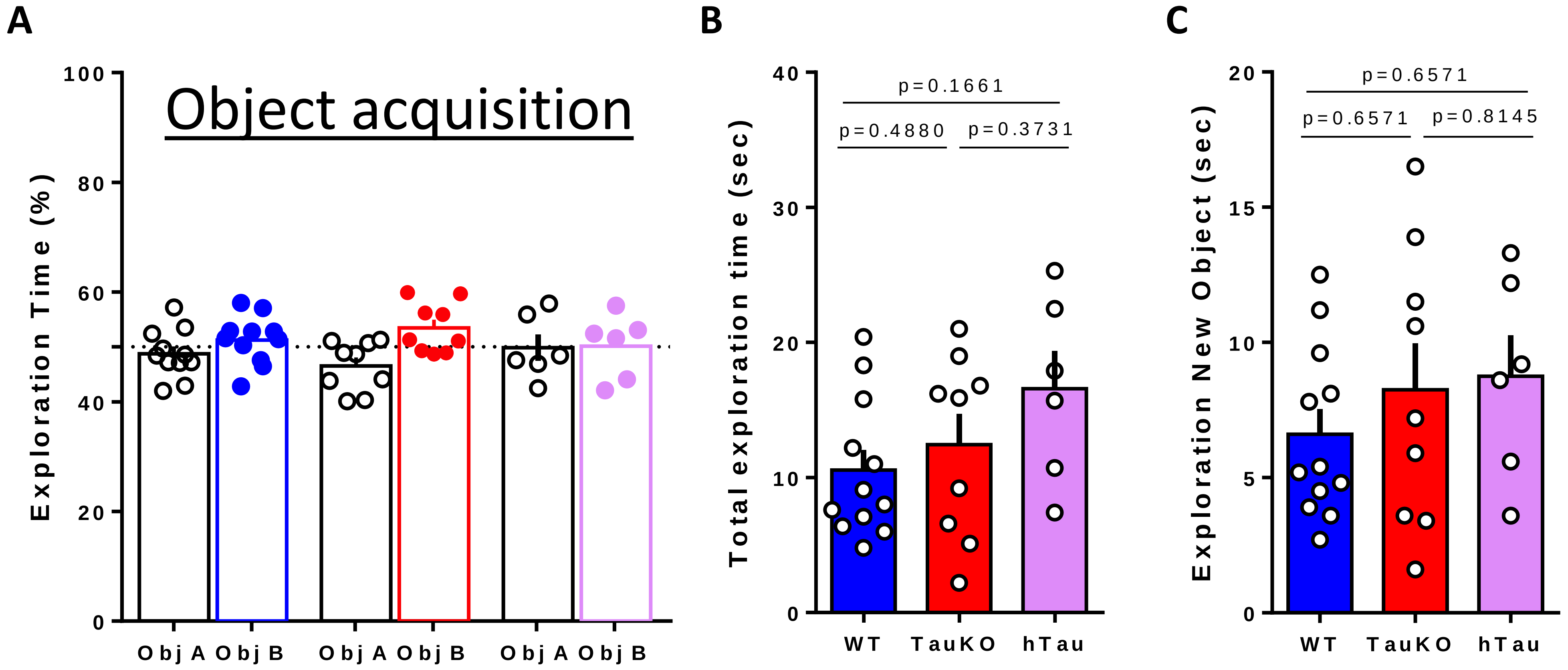

SUPPLEMENTARY FIGURE 1. Novel Object Recognition test data

Behavioral test performed with 15-19 weeks old WT, TauKO and hTau mice. **(A)** Exploration time of object A and B during the training phase showing no object preference. Time expressed in percentage. **(B)** Total exploration of familiar and novel objects during the test phase. Time expressed in seconds. **(C)** Total exploration of novel object during the test phase. Time expressed in seconds. Data are representative of at least two independent experiments.

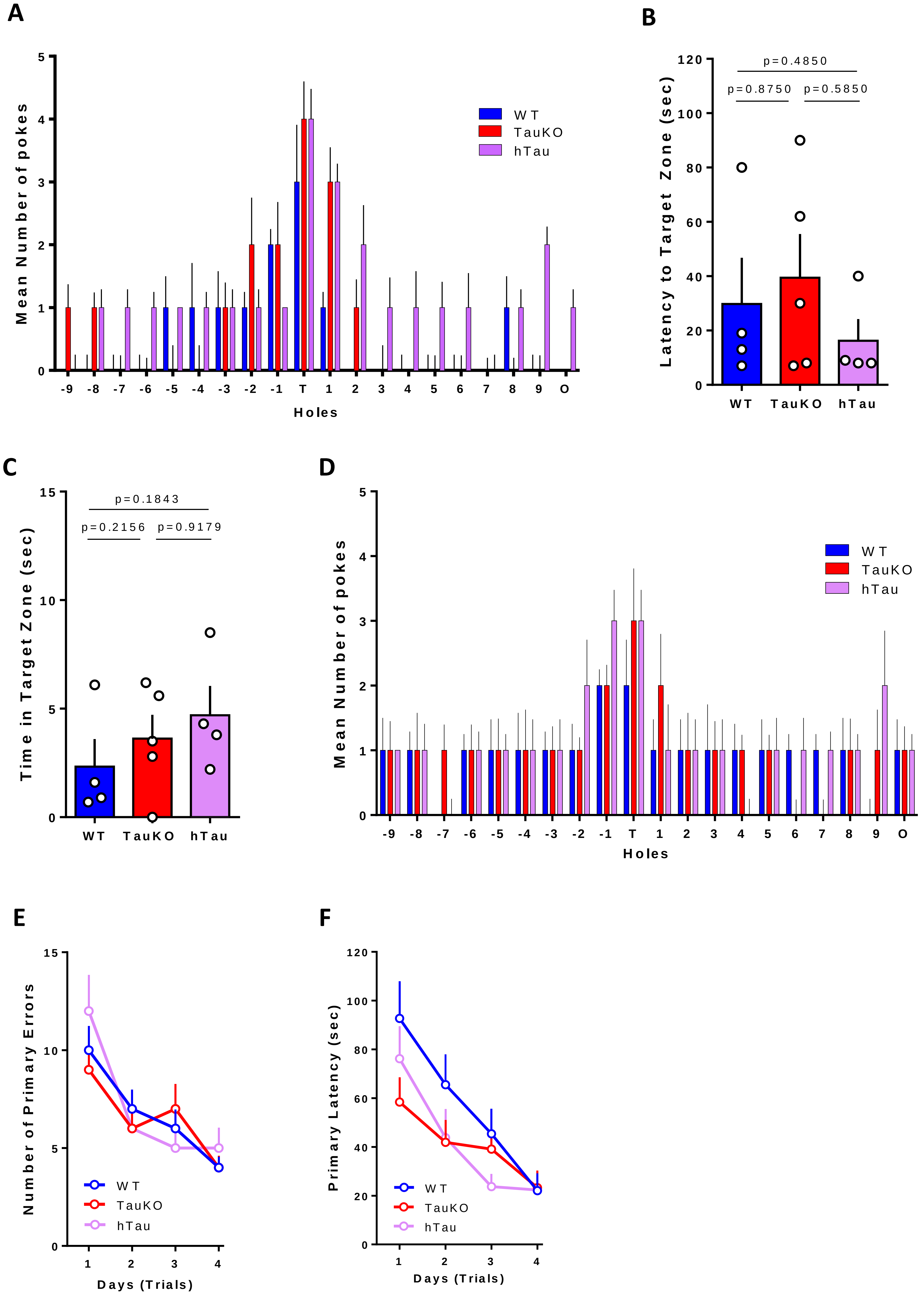

SUPPLEMENTARY FIGURE 2. Barnes maze test data

Behavioral test performed with 15-19 weeks old WT, TauKO and hTau mice. **(A)** Histograms represent the mean number of nose pokes in each hole of the Barnes Maze during the test (Probe day 5, 24 hours after the last training trial). **(B-D)** Results from probe day 12, 8 days after the last training trial. **(B)** Latency to find and **(C)** time in the target zone. **(D)** Histograms represent the mean number of nose pokes in each hole of the Barnes Maze during the test (Probe day 12). **(E)** Number of primary errors and **(F)** primary latency to find the target zone in 4 different trials/day, for 4 days during the acquisition/training phase of the Barnes maze test. T: target hole, O: opposite hole to target. Data are representative of at least two independent experiments.
